# Increased risk of pulmonary hypertension following premature birth

**DOI:** 10.1186/s12887-019-1665-6

**Published:** 2019-08-17

**Authors:** Estelle Naumburg, Lars Söderström

**Affiliations:** 1 Department of Clinical Science, Pediatrics, Umeå University, Umeå, Sweden; 2 Unit of Research, Education and Development, Östersund Hospital, Östersund, Sweden; 3 Pediatrics department, Östersund Hospital, SE-831 83 Östersund, Sweden

**Keywords:** Bronchopulmonary dysplasia, Lung disease, Preterm birth, Pulmonary hypertension

## Abstract

**Background:**

Pulmonary hypertension (PAH) among children and adults has been linked to premature birth, even after adjustments for known risk factors such as congenital heart disease and chronic lung disease. The aim of this population-based registry study was to assess the risk of PAH following exposure to premature birth and other factors in the decades when modern neonatal care was introduced and survival rates increased.

**Methods:**

Data on pulmonary hypertension and perinatal factors were retrieved from population-based governmental and national quality registers. Cases were adults and children over five years of age with pulmonary hypertension born from 1973 to 2010 and individually matched to six controls by birth year and delivery hospital. Conditional multiple logistic regression was performed to assess the risk of pulmonary hypertension following premature birth and to adjust for known confounding factors for the total study population and for time of birth, grouped into five-year intervals.

**Results:**

In total, 128 cases and 768 controls were included in the study group. Preterm birth was over three times more common among cases (21%) than among controls (6%). The overall adjusted risk of pulmonary hypertension was associated with premature birth, OR = 4.48 (95% CI; 2.10–9.53). Maternal hypertension, several neonatal risk factors and female gender were independently associated with PAH when potential confounders were taken into account. For each five-year period, the risk of PAH following premature birth increased several times for children born in the 2000s and later, OR = 17.08 (95% CI 5.60–52.14).

**Conclusions:**

Preterm birth, along with other factors, significantly contributes to PAH. PAH following premature birth has increased over the last few decades. Our study indicates that new, yet unknown factors may play a role in the risk of preterm-born infants developing PAH later in life.

## Background

Preterm birth has previously been linked to pulmonary arterial hypertension (PAH) in children and adults [1]. PAH is a multifactorial disease and may have several origins, such as congenital heart disease (CHD); chronic lung disease (CLD), such as bronchopulmonary dysplasia (BPD); genetic predepositions; or vascular growth factors. Surfactant and antenatal corticosteroid treatments have been in clinical use in Sweden since the early 1990s. This has reduced the incidence of respiratory morbidity and mortality among children born prematurely [2, 3]. However, long-term impairments of lung function, airway obstruction, and structural impairments of gas transfer and pulmonary function remain [4, 5]. In previous studies, we found that the risk of PAH several years after birth for children born prematurely has increased over time, even when known risk factors such as CLD, BPD and CHD are adjusted for [6, 7]. The aim of this study was to assess the risk of PAH following exposure to preterm birth and other known risk factors over several decades and to assess the impact of the introduction of external surfactant and antenatal corticosteroids.

## Methods

This population-based national case-control registry study assessed neonatal risk factors for children and young adults with pulmonary hypertension compared to healthy controls. The study is based on registry data, and individual informed consent from each participant is not required due to a waiver from the ethical committee and national guidelines.

### Study population

All children aged five years or older and adults who were born between 1973 and 2010 and who were registered in the population-based Swedish Medical Birth Register were included in this case-control study. Cases were all diagnosed with PAH; those who were born in 1973–1996 were retrieved from the Swedish Pulmonary Arterial Hypertension Registry (SPAHR), and those who were born in 1993–2010 were retrieved from the Swedish registry of Congenital heart disease (SWEDCON). All cases retrieved from SPAHR were diagnosed according to the Dana Point Classification using right heart catheterization, and the cases retrieved from SWEDCON were diagnosed using either right heart catheterization and/or transthoracic Doppler echocardiography. The registries and retrieval of cases and controls are described in previously published studies [6–8].

Six controls without pulmonary hypertension were matched to each case by year of birth and hospital. Cases who were not born in Sweden were excluded.

A national registration number is assigned at birth to every child born in Sweden.

### Exposure data

Maternal factors during pregnancy (age, hypertension, smoking, pregnancy), neonatal data (premature birth, acute pulmonary disease, BPD, congenital diaphragmatic hernia, CHD, chronic pulmonary disease, gender, first born status, chromosomal abnormalities, large for gestational age, persisting pulmonary hypertension of the newborn, small for gestational age, APGAR score at one and five minutes and birth weight) were retrieved from the Swedish Medical Birth Register using the International Classification of Diseases ICD-9 or ICD-10 codes [9]. Preterm birth was defined as birth prior to 37 weeks of gestation.

Linkages between governmental and national quality-based registries was possible with national registration numbers, which were used for the retrieval of exposure information for both cases and controls [10] .

### Statistical methods

The association between preterm birth prior to 37 weeks of gestation and PAH for the whole period (1973–2010) was calculated by conditional logistic regression and adjusted for confounding factors.

To assess the association between preterm birth and PAH over time, we subgrouped the study population into five-year intervals based on the year of birth. We then calculated the risk of PAH following premature birth for each group and adjusted for confounding factors. Exposure to potential confounders is described in Table 1 and was used in the multivariable regression model.

**Table 1:** Neonatal characteristics of the total study population and for each five-year birth interval

Variable	Status	1973–1977	1978–1982	1983–1987	1988–1992	1993–1997	1998–2002	2003–2007	2008–2010	Total (*N*)	%
Total number of cases	Case	21	18	16	4	14	12	17	26	128	
Total number of controls	Control	126	108	96	24	84	72	102	156	768	
Maternal age (years)	Case	25,0	27,1	28,8	24,8	29,5	31,9	31,5	32,3	29,3	
	Control	24,4	27,5	28,9	26,3	29,4	29,0	30,8	29,9	28,7	
	*p*-value	0.2306	0.7756	0.9704	0.4878	0.9463	0.0567	0.611	0.0251	0.2905	
Maternal hypertension	Case	2	4	0	0	3	1	2	3	15	12%
	Control	11	8	5	1	1	1	3	0	37	5%
	p-value	1	0.0696	1	1	0.0087	0.2669	0.144	1	0.0064	
Maternal smoking	Case	–	1	2	0	2	0	1	2	8	6%
	Control	–	2	24	5	9	6	11	6	63	8%
	p-value	–	1	0.5097	1	0.6324	0.58	1	0.2671	0.7108	
Premature birth	Case	1	1	0	0	0	2	5	18	27	21%
	Control	4	4	5	4	8	5	2	15	47	6%
	p-value	0.2045	0.5467	0.0860	1.0000	0.1902	0.0044	8.82E-05	< 0.0001	< 0.0001	
Acute neonatal pulmonary disease	Case	1	1	0	0	2	3	5	15	27	21%
	Control	4	6	4	1	6	6	3	13	43	6%
	*p*-value	0.5427	1.0000	1.0000	1.0000	0.3200	0.1146	0.0014	< 0.0001	< 0.0001	
Bronchopulmonary dysplasia	Case	0	0	0	0	1	1	2	10	14	11%
	Control	2	0	0	0	0	0	0	0	2	0%
	*p*-value	1.0000	1.0000	1.0000	1.0000	0.1429	0.1429	0.0194	< 0.0001	< 0.0001	
Congenital diaphragmatic hernia	Case	0	0	0	1	3	0	0	2	6	1%
	Control	0	0	0	0	1	0	0	0	1	0%
	*p*-value	1.0000	1.0000	1.0000	0.1429	0.0087	1.0000	1.0000	0.0197	< 0.0001	
Congenital heart disease	Case	2	3	3	2	2	2	5	4	23	18%
	Control	2	2	0	0	3	1	0	2	10	1%
	*p*-value	0.0979	0.0207	0.0026	0.0159	0.1476	0.0522	3.39E-05	0.0041	< 0.0001	
Chronic pulmonary disease	Case	0	0	0	0	1	1	2	11	15	12%
	Control	2	0	0	0	0	0	0	1	3	0%
	*p*-value	1.0000	1.0000	1.0000	1.0000	0.1429	0.1429	0.0194	< 0.0001	< 0.0001	
First-born child	Case	9	8	7	4	8	2	5	10	53	41%
	Control	56	46	43	9	34	23	53	82	316	41%
	*p*-value	0.6429	0.8001	0.1379	0.2734	0.2494	0.1199	0.7872	0.5252	1.000	
Female sex	Case	15	9	11	3	5	10	6	13	72	56%
	Control	56	46	43	9	34	23	53	82	346	45%
	*p*-value	0.0324	0.6132	0.1051	0.2850	1.0000	0.0011	0.2950	0.8349	0.0215	
Chromosomal abnormalities	Case	1	1	1	1	2	1	3	0	10	8%
	Control	0	0	0	0	0	0	0	1	1	0%
	*p*-value	0.1428	0.1428	0.1428	0.1428	0.0191	0.1428	0.0025	1.0000	< 0.0001	

Maximum-likelihood estimates of the odds ratio (OR) and 95% confidence interval (CI) were obtained. SAS version 9.4 (SAS Institute, Inc., Cary, NC, USA) was used to fit the conditional logistic model to our 1:6 matched case-control data.

The study was approved by the regional ethics committee of Umeå University (D2011–396-31 M).

## Results

### The total study group

Overall, 128 cases, children and adults with PAH, were included in the study and individually matched to six controls each (*N* = 768). The median birth year was 1994, with an interquartile range (IQR) of 26 years.

**Fig. 1:**
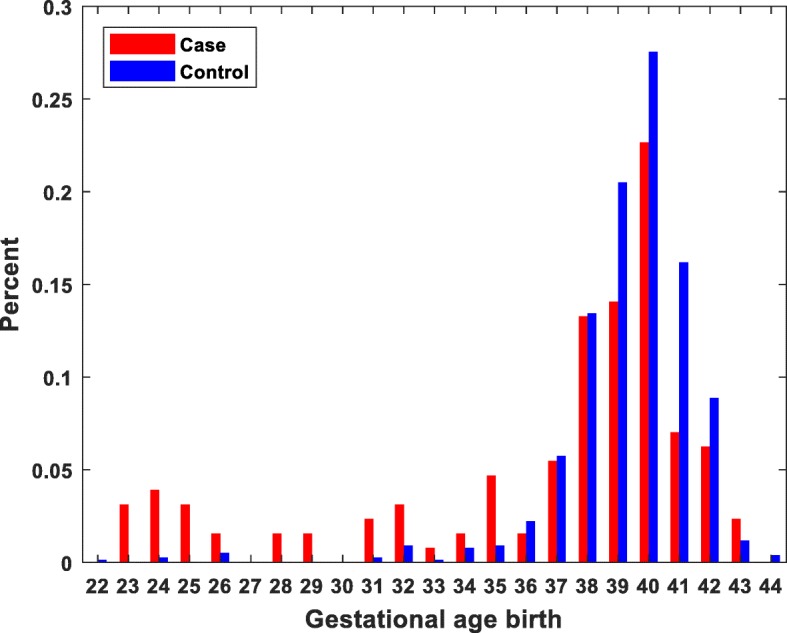
Gestational age at birth among cases and controls

Preterm birth was over three times more common among cases (6%) than among controls (21%) for the total study group (Table 1, Fig. 1). (Table 1, Fig. 1). Maternal hypertension and several other neonatal characteristics were more common among cases than controls (Table 1). Acute and chronic pulmonary disease, BPD, congenital diaphragmic hernia, CHD, and female gender were more common for cases than controls (Table 1). Chromosomal abnormalities were present in ten of the cases (8%) but only one of the controls (Table 1).

### Results by birth year intervals

Birth weight was generally lower among cases in all age groups and even more common among those in the more recently born groups (Table 2). Apgar scores were lower among cases than controls born in more recent years (Table 2).

**Table 2:** Birth weight and Apgar scores per five-year interval of the study population born 1973–2010

		Birth weight (grams)			Apgar 1 min			Apgar 5 min		
Birth year	Status	Number	Mean	Missing info	No	Mean	Missing info	No	Mean	Missing info
1973–1977	Case	21	3070.0	0	21	9.0	0	13	9.5	8
	Control	126	3477.4	0	125	9.0	1	107	9.4	19
	Total/*p*-value	147	0.0037		146	0.6129		120	0.6295	
1978–1982	Case	18	3491.1	0	18	8.7	0	17	9.0	1
	Control	107	3491.2	1	106	8.8	2	96	9.0	12
	Total/*p*-value	125	0.9357		124	0.4550		113	0.5960	
1983–1987	Case	16	3269.7	0	16	8.4	0	15	9.5	1
	Control	95	3521.2	1	94	8.7	2	92	9.8	4
	Total/*p*-value	111	0.1860		110	0.6410		107	0.2436	
1988–1992	Case	4	3030.0	0	4	8.8	0	4	7.5	0
	Control	24	3416.0	0	24	9.0	0	24	9.7	0
	Total/*p*-value	28	0.2001		28	0.3104		28	0.0511	
1993–1997	Case	13	2801.2	1	12	8.2	2	12	8.5	2
	Control	84	3432.0	0	84	8.7	0	83	9.8	1
	Total/*p*-value	97	0.0465		96	0.2767		95	0.0008	
1998–2002	Case	12	2668.3	0	11	7.4	1	11	8.8	1
	Control	70	3595.0	2	71	8.8	1	71	9.8	1
	Total/*p*-value	82	0.0100		82	0.0078		82	0.0002	
2003–2007	Case	17	2474.1	0	17	6.9	0	17	8.5	0
	Control	102	3587.9	0	100	8.8	2	100	9.8	2
	Total/*p*-value	119	0.0010		117	0.0004		117	0.0011	
2008–2010	Case	25	1923.4	1	26	5.6	0	26	7.5	0
	Control	156	3441.3	0	153	8.5	3	153	9.5	3
	Total/*p*-value	181	< 0.0001		179	< 0.0001		179	< 0.0001	

### Risk estimations

Preterm birth was associated with an increased risk of PAH for the total study group over the whole study period, OR = 4.6 (95% CI = 2.2–9.8) (Table 3). Maternal hypertension, congenital diaphragmatic herniation, congenital heart defects, chromosomal abnormalities, PPHN and female sex were independently associated with PAH when potential confounders were taken into account (Table 3).

**Table 3:** Risk factors associated with pulmonary hypertension in children born 1973–2010

Variable	Cases *N* = 128	Controls *N* = 768 (missing)	Odds ratio	95% confidence interval	*p*-value
Premature birth	37	47	4.48	2.10–9.52	0.0001
Female sex	72	346	1.69	1.06–2.71	0.0277
Small for gestational age	7	13	4.06	1.14–14.49	0.0310
Large for gestational age	6	21	3.53	1.21–10.31	0.0212
Chronic pulmonary disease	15	3	13.07	2.21–77.19	0.0046
Persistent pulmonary hypertension at birth	27	10	15.01	5.57–40.44	0.0000
Congenital heart defect	23	10	10.12	3.94–25.99	0.0000
Chromosomal abnormalities	10	1	67.71	4.70–974.90	0.0020
Maternal hypertension	15	37	2.70	1.12–6.54	0.0276

**Fig. 2:**
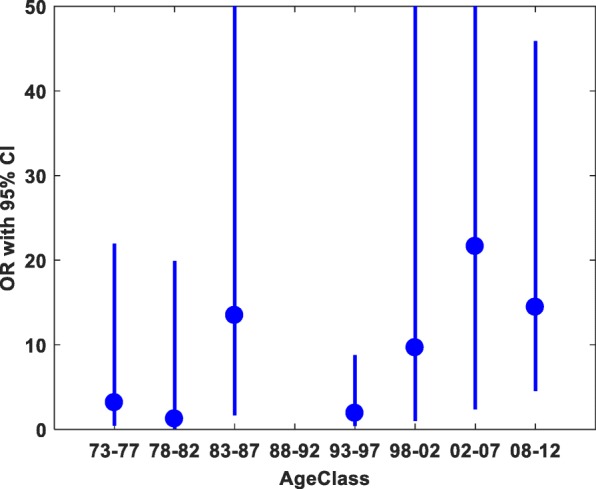
Pulmonary hypertension associated with premature birth for the study population in five-year subgroups

Being born premature in 1983–87, 2003–07 and 2008–15 was significantly associated with PAH later in life, although with wide confidence intervals (Fig. 2).

## Discussion

Surviving preterm birth was associated with PAH among children older than five years and adults. This risk did not alter after adjustment for known risk factors. A history of pulmonary neonatal diseases was also associated with pulmonary hypertension when growing up. The risk of developing PAH was increased for several five-year birth cohorts, although the confidence intervals were large. Being born prematurely was much more common among cases belonging to later birth cohorts.

In Sweden, preterm birth occurs in 6% of infants each year. Children who were born in the 1970s seldom survived a premature birth at gestational ages at which we now expect almost 100% survival rates [11]. Neonatal deaths have decreased from nearly 8% in 1973 to 1.6% in Sweden today [11]. This study thus covers a period of great advances in neonatal care. Factors such as antenatal corticosteroid treatment for women at risk of preterm delivery and surfactant for newborns have been proven to induce fetal pulmonary maturation and reduce respiratory morbidity and mortality [2, 3, 12–14]. Surfactant and antenatal corticosteroids have been in clinical use in Sweden since the early 1990s and 1980s, respectively. In our study, the risk of developing pulmonary hypertension was greater for a child born in the 2000s than for one born in the 1970s or 1980s. This difference can be explained by the greater survival rates due to advances in neonatal care. The risk of developing PAH for a child born premature during the 1970s and 1980s who reached adulthood must be regarded as less likely than today, mainly because many children did not survive the neonatal period during these years.

The clinical pattern of BPD has changed during the surfactant era, affecting smaller and more immature infants. The overall incidence of any form of acute lung disease in a newborn is approximately 3%, and it increases with decreasing gestational age and birthweight [15]. Reduced pulmonary function has been associated with low birthweights and preterm birth, and in a recent study, an association with prenatal exposures was discussed [16]. Airflow limitation, along with impaired exercise capacity and systolic function of the right ventricle, is present in adolescents and young adults who survive preterm birth, even in cases of mild lung diseases [17–21].

Premature birth has been reported by others as common among children with PAH (14–21.8%) and even more common when a pulmonary disease is related to the PAH diagnosis [22, 23]. Premature birth was present in cases as well as controls. The overall rate of 6% for premature birth in Sweden has not changed for several decades [9]. The proportion of premature births among the controls was in line with what was expected. In our study, premature birth was more than three times higher among cases than controls, and in the most recent birth year cohorts, the difference was even greater. Our study strengthens the hypothesis that exposure to premature birth increases the risk of PAH, but the underlying reasons for this effect are still unknown. Several factors, in addition to exposure to premature birth, may influence the risk of PAH as growing up. The study group was too small to assess whether there is an association between lower gestational age and premature birth.

Angiogenesis has been shown to be necessary for alveolarization during normal lung development [24]. The expression of growth factors, as well as the lung response to hypoxia, has been linked to lung diseases such as persistent pulmonary hypertension of the newborn (PPHN) and bronchopulmonary dysplasia (BPD) [25]. Pulmonary vascular growth during fetal and neonatal life is dependent on endothelial cells, numerous growth factors and cytokines, of which vascular endothelial growth factors are the most important [24, 26–30]. Vascular growth is driven by endothelial vascular cells, forming stable connections and cellular rearrangements during sprouting, anastomosis, lumen formation, and functional remodeling of the vascular network [31, 32]. However, animal studies show that once blood flow is established, the pruning and growth ends [33]. The transition to extrauterine circulation involves increasing oxygen saturation to nearly normal levels and establishing an 8- to 10-fold increase in pulmonary blood flow [34, 35]. Altered pulmonary artery thickness and stiffness have been reported in prematurely born children, indicating that vascular impairment is part of the BPD pathology [36, 37].

We speculate that the discontinuation of normal lung vascularization in premature birth has an adverse impact on the vascular development of the infant’s lungs and on future growth. This may induce stress on the myocardium, causing PAH to occur later in life as the individual is exposed to other factors that further impair heart function. Evidence of echocardiographic myocardial changes has recently been found in preterm children at one year of age, but further studies of pulmonary vascular maturation in relation to cardiac function are needed [38]. Medical treatments that influence pulmonary vascular growth may be the next step in neonatal care advancement.

Matching cases and controls by year and birth hospital reduced the risk of selection bias due to differences in medical care and survival rates. To increase power, we choose to match six controls to each case.

Cardiovascular malformations, as well as chromosomal abnormalities, include heterogeneous conditions; they are more common among preterm infants than term-born infants and are also known risk factors for PAH [39, 40]. By adjusting for CHD and chromosomal abnormalities, we ruled out this confounding factor in our study. To test this hypothesis, we performed additional analyses excluding children with chromosomal abnormalities or excluding the variable chromosomal abnormalities; these exclusions did not alter the results.

There is always the risk of the misclassification of diagnosis when using registers. We believe that the potential bias of cases is small in our study as all adult cases were retrieved from the SPAHR, which includes patients according to the Dana Point classification [41, 42], and the SWEDCON register, which recently has been validated and showed good concordance between register data and medical records [8].

## Conclusions

Preterm birth, along with other factors, significantly contributes to the development of PAH. Previously, CHD, pulmonary diseases and other factors have been linked to PAH in children and young adults who were born preterm. By adjusting for previously known risk factors, our study indicates that new, yet undefined and unknown factors may play a role in the risk of PAH development in later life among those born preterm. In this paper, we discuss some hypotheses to be tested in future studies.

## Data Availability

The data that support the findings of this study are available from the Swedish Society for Pulmonary Hypertension, the Swedish Registry of Congenital Heart Disease and the Swedish Medical Birth Register. Restrictions may apply to the availability of these data, which were used under license for the current study and so are not publicly available. Data are, however, available from the authors upon reasonable request and with permission from the Swedish Society for Pulmonary Hypertension, the Swedish Registry of Congenital Heart Disease and the Swedish Medical Birth Register.

## Abbreviations

APA: Appropriate for gestational age
BPD: Bronchopulmonary dysplasia
CHD: Congenital heart disease
CI: Confidence interval
CLD: Chronic lung disease
CPAP: Continuous positive airway pressure
EPCC: European Pediatric Cardiology Codes
ICD-10 codes: International Classification of Diseases (ICD-10 codes)
LGA: Large for gestational age
OR: Odds ratio
PAH: Pulmonary arterial hypertension
PPHN: Persistent pulmonary hypertension as a neonate
SD: Standard deviation
SGA: Small for gestational age
SWEDCON: Swedish Congenital Heart Defect Register

## References

[1] Abman Steven H. (2010). Pulmonary hypertension in children: A historical overview. Pediatric Critical Care Medicine.

[2] Lee K, Khoshnood B, Wall SN, Chang Y, Hsieh HL, Singh JK (1999). Trend in mortality from respiratory distress syndrome in the United States, 1970-1995. J Pediatr.

[3] Crowley P, Chalmers I, Keirse MJ (1990). The effects of corticosteroid administration before preterm delivery: an overview of the evidence from controlled trials. Br J Obstet Gynaecol.

[4] Koivisto M, Marttila R, Kurkinen-Raty M, Saarela T, Pokela ML, Jouppila P (2004). Changing incidence and outcome of infants with respiratory distress syndrome in the 1990s: a population-based survey. Acta Paediatr.

[5] Halvorsen T, Skadberg BT, Eide GE, Roksund OD, Markestad T (2006). Better care of immature infants; has it influenced long-term pulmonary outcome?. Acta Paediatr.

[6] Naumburg E, Axelsson I, Huber D, Soderstrom L (2015). Some neonatal risk factors for adult pulmonary arterial hypertension remain unknown. Acta Paediatr.

[7] Naumburg E, Soderstrom L, Huber D, Axelsson I (2017). Risk factors for pulmonary arterial hypertension in children and young adults. Pediatr Pulmonol.

[8] Bodell A, Björkhem G, U. T, Naumburg E. National quality register of congenital heart diseases – Can we trust the data? J Congenit Heart Dis 2017;1(11).

[9] Welfare SNBoHa. Pregnancies, Deliveries and Newborn Infants The Swedish Medical Birth Register 1973–2009 Assisted Reproduction, treatment 1991–2008. OFFICIAL STATISTICS OF SWEDEN Statistics – Health and Medical Care. Socialstyrelsen; 2010. Contract No.: 2011–3-19.

[10] Ludvigsson JF, Almqvist C, Bonamy AK, Ljung R, Michaelsson K, Neovius M (2016). Registers of the Swedish total population and their use in medical research. Eur J Epidemiol.

[11] Fellman V, Hellstrom-Westas L, Norman M, Westgren M, Kallen K, Lagercrantz H (2009). One-year survival of extremely preterm infants after active perinatal care in Sweden. JAMA..

[12] Liechty EA, Donovan E, Purohit D, Gilhooly J, Feldman B, Noguchi A (1991). Reduction of neonatal mortality after multiple doses of bovine surfactant in low birth weight neonates with respiratory distress syndrome. Pediatrics..

[13] Halliday Henry L. (1995). Overview of Clinical Trials Comparing Natural and Synthetic Surfactants. Neonatology.

[14] Ehrenkranz RA, Walsh MC, Vohr BR, Jobe AH, Wright LL, Fanaroff AA (2005). Validation of the National Institutes of Health consensus definition of bronchopulmonary dysplasia. Pediatrics..

[15] Rubaltelli FF, Bonafe L, Tangucci M, Spagnolo A, Dani C (1998). Epidemiology of neonatal acute respiratory disorders. A multicenter study on incidence and fatality rates of neonatal acute respiratory disorders according to gestational age, maternal age, pregnancy complications and type of delivery. Italian Group of Neonatal Pneumology. Biol Neonate.

[16] Schultz ES, Hallberg J, Andersson N, Thacher JD, Pershagen G, Bellander T (2018). Early life determinants of lung function change from childhood to adolescence. Respir Med.

[17] Baraldi Eugenio, Carraro Silvia, Filippone Marco (2009). Bronchopulmonary dysplasia: Definitions and long-term respiratory outcome. Early Human Development.

[18] Lewandowski AJ, Bradlow WM, Augustine D, Davis EF, Francis J, Singhal A (2013). Right ventricular systolic dysfunction in young adults born preterm. Circulation..

[19] Smith LJ, van Asperen PP, McKay KO, Selvadurai H, Fitzgerald DA (2008). Reduced exercise capacity in children born very preterm. Pediatrics..

[20] Vollsaeter M, Roksund OD, Eide GE, Markestad T, Halvorsen T (2013). Lung function after preterm birth: development from mid-childhood to adulthood. Thorax..

[21] Skromme Kaia, Leversen Katrine Tyborg, Eide Geir Egil, Markestad Trond, Halvorsen Thomas (2015). Respiratory illness contributed significantly to morbidity in children born extremely premature or with extremely low birthweights in 1999-2000. Acta Paediatrica.

[22] Berger RM, Beghetti M, Humpl T, Raskob GE, Ivy DD, Jing ZC (2012). Clinical features of paediatric pulmonary hypertension: a registry study. Lancet..

[23] del Cerro Marin MJ, Sabate Rotes A, Rodriguez Ogando A, Mendoza Soto A, Quero Jimenez M, Gavilan Camacho JL (2014). Assessing pulmonary hypertensive vascular disease in childhood. Data from the Spanish registry. Am J Respir Crit Care Med.

[24] Jakkula M, Le Cras TD, Gebb S, Hirth KP, Tuder RM, Voelkel NF (2000). Inhibition of angiogenesis decreases alveolarization in the developing rat lung. Am J Physiol Lung Cell Mol Physiol..

[25] Abman SH (2010). Impaired vascular endothelial growth factor signaling in the pathogenesis of neonatal pulmonary vascular disease. Adv Exp Med Biol.

[26] Abman SH (2001). Bronchopulmonary dysplasia: "a vascular hypothesis". Am J Respir Crit Care Med.

[27] Tsao PN, Wei SC (2013). Prenatal hypoxia downregulates the expression of pulmonary vascular endothelial growth factor and its receptors in fetal mice. Neonatology..

[28] Ivy DD, le Cras TD, Parker TA, Zenge JP, Jakkula M, Markham NE (2000). Developmental changes in endothelin expression and activity in the ovine fetal lung. Am J Physiol Lung Cell Mol Physiol.

[29] Bhatt AJ, Pryhuber GS, Huyck H, Watkins RH, Metlay LA, Maniscalco WM (2001). Disrupted pulmonary vasculature and decreased vascular endothelial growth factor, Flt-1, and TIE-2 in human infants dying with bronchopulmonary dysplasia. Am J Respir Crit Care Med.

[30] Wallace B, Peisl A, Seedorf G, Nowlin T, Kim C, Bosco J (2018). Anti-sFlt-1 therapy preserves lung alveolar and vascular growth in antenatal models of bronchopulmonary dysplasia. Am J Respir Crit Care Med.

[31] Szymborska Anna, Gerhardt Holger (2017). Hold Me, but Not Too Tight—Endothelial Cell–Cell Junctions in Angiogenesis. Cold Spring Harbor Perspectives in Biology.

[32] Neto F, Klaus-Bergmann A, Ong YT, Alt S, Vion AC, Szymborska A, et al. YAP and TAZ regulate adherens junction dynamics and endothelial cell distribution during vascular development. Elife. 2018;7. 10.7554/eLife.31037 PMC5814147 29400648

[33] Chen Q, Jiang L, Li C, Hu D, Bu JW, Cai D (2012). Haemodynamics-driven developmental pruning of brain vasculature in zebrafish. PLoS Biol.

[34] Hooper SB, Te Pas AB, Lang J, van Vonderen JJ, Roehr CC, Kluckow M (2015). Cardiovascular transition at birth: a physiological sequence. Pediatr Res.

[35] McVea Steven, McGowan Michael, Rao Bharathi (2018). How to use saturation monitoring in newborns. Archives of disease in childhood - Education & practice edition.

[36] Wagner BD, Babinec AE, Carpenter C, Gonzalez S, O'Brien G, Rollock K (2018). Proteomic profiles associated with early echocardiogram evidence of pulmonary vascular disease in preterm infants. Am J Respir Crit Care Med.

[37] Sehgal A, Gwini SM, Menahem S, Allison BJ, Miller SL, Polglase GR (2019). Preterm growth restriction and bronchopulmonary dysplasia: the vascular hypothesis and related physiology. J Physiol.

[38] Levy PT, Patel MD, Choudhry S, Hamvas A, Singh GK (2018). Evidence of echocardiographic markers of pulmonary vascular disease in asymptomatic infants born preterm at one year of age. J Pediatr.

[39] Tanner K, Sabrine N, Wren C (2005). Cardiovascular malformations among preterm infants. Pediatrics..

[40] Harries C, Armstrong I (2012). A review of the management of pulmonary arterial hypertension associated with congenital heart disease. Eur J Cardiovasc Nurs.

[41] Simonneau Gerald, Gatzoulis Michael A., Adatia Ian, Celermajer David, Denton Chris, Ghofrani Ardeschir, Gomez Sanchez Miguel Angel, Krishna Kumar R., Landzberg Michael, Machado Roberto F., Olschewski Horst, Robbins Ivan M., Souza Rogiero (2013). Updated Clinical Classification of Pulmonary Hypertension. Journal of the American College of Cardiology.

[42] Galie N, Hoeper MM, Humbert M, Torbicki A, Vachiery JL, Barbera JA (2009). Guidelines for the diagnosis and treatment of pulmonary hypertension: the task force for the diagnosis and treatment of pulmonary hypertension of the European Society of Cardiology (ESC) and the European Respiratory Society (ERS), endorsed by the International Society of Heart and Lung Transplantation (ISHLT). Eur Heart J.

